# Supplementary material for: Automated HER2 Scoring with Uncertainty Quantification Using Lensfree Holography and Deep Learning
Source: BME Front. 2026 Jun 16;7:0278. doi: 10.34133/bmef.0278 (PMC13269775; doi:10.34133/bmef.0278)
Supplement: Supplementary 1 — Figs. S1 to S7 Movie S1 [file bmef.0278.f1.zip › Lensless_HER2_SI_vR vF_TBS CLEAN.pdf]

Supplementary Materials for

Automated HER2 scoring with uncertainty quantification using lensfree holography and deep learning

Che-Yung Shen[1,2,3†], Xilin Yang[1,2,3†], Yuzhu Li[1,2,3†], Leon Lenk[4], Zixiang Ji[4], and Aydogan Ozcan[1,2,3*]

[1]Electrical and Computer Engineering Department, University of California, Los Angeles, CA, 90095, USA
[2]Bioengineering Department, University of California, Los Angeles, CA, 90095, USA
[3]California NanoSystems Institute (CNSI), University of California, Los Angeles, CA, 90095, USA
[4]Department of Computer Science, University of California, Los Angeles, CA, 90095, USA

†These authors contributed equally to the work
*Correspondence to: ozcan@ucla.edu

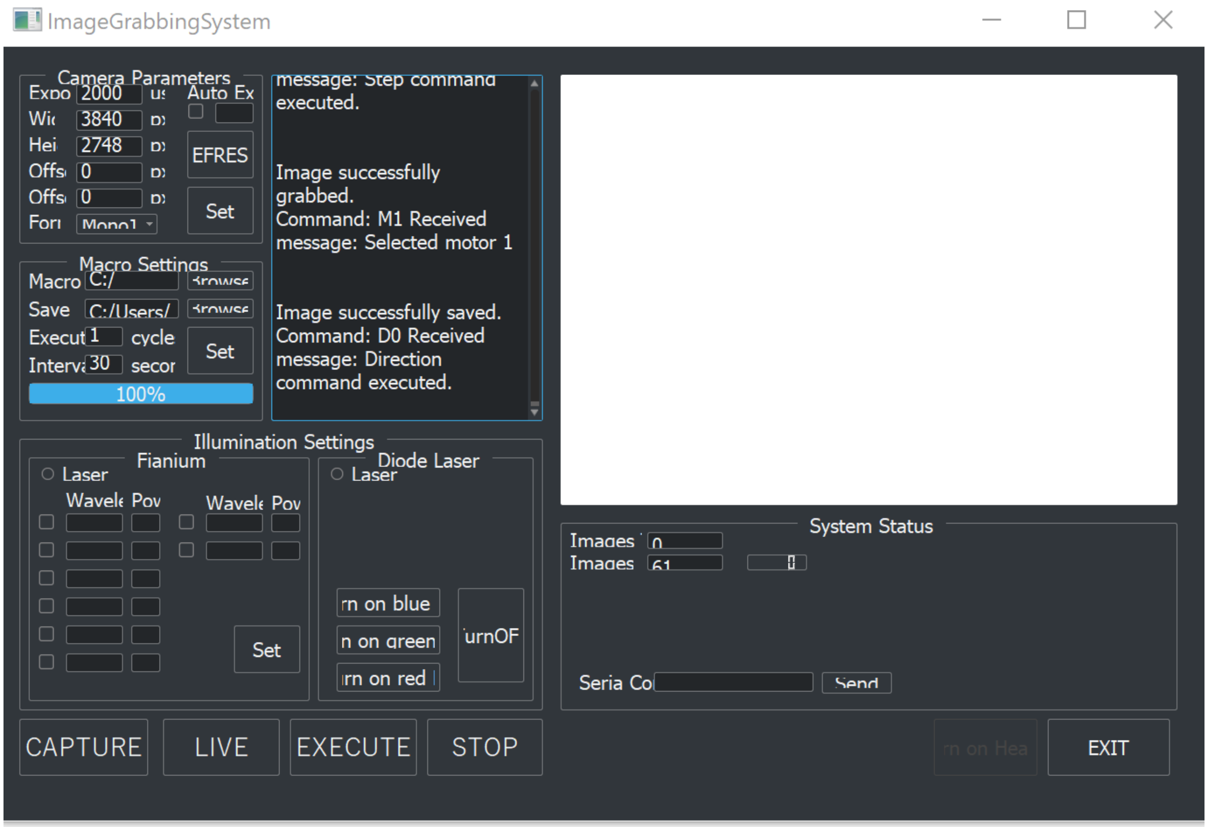

Fig. S1. Graphical user interface (GUI) of the controlling program. Users can adjust the illumination, image sensor, and scan settings through this GUI.

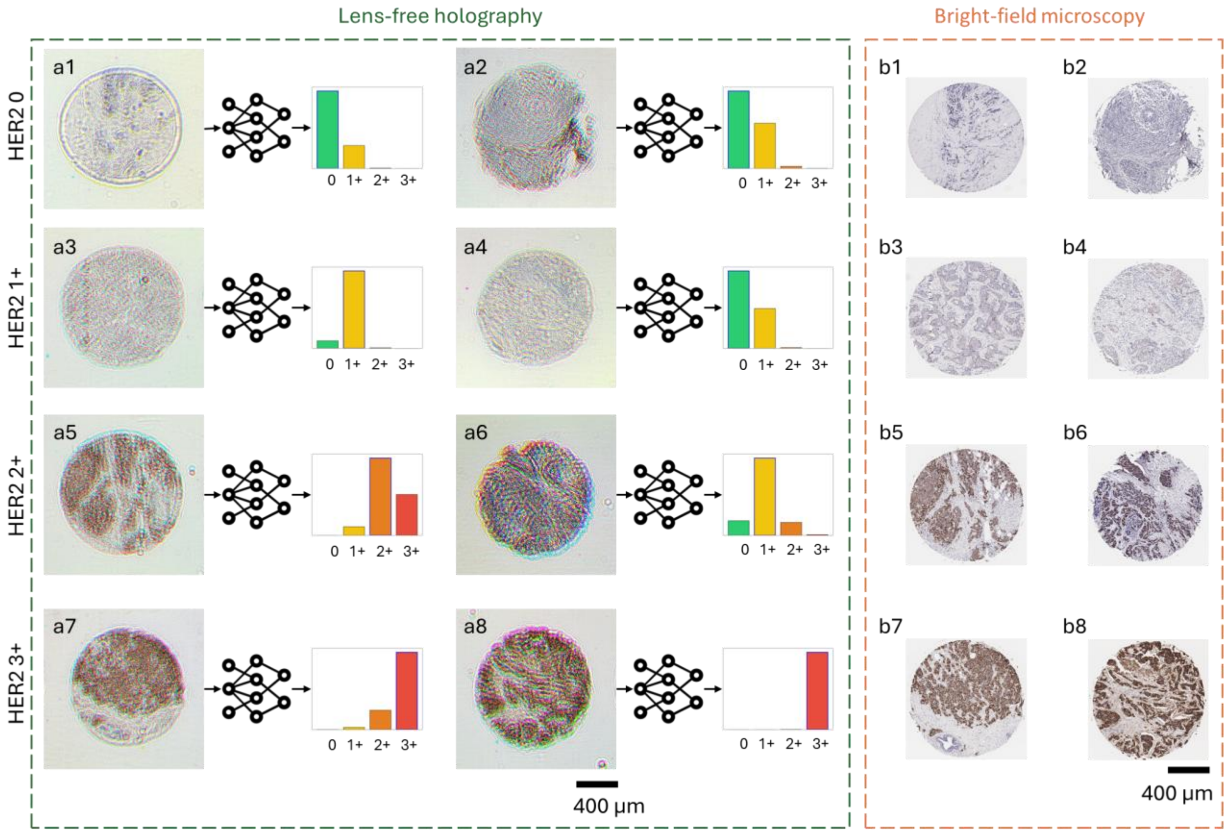

Fig. S2. Exemplified images of the lensfree holograms and the corresponding brightfield microscopy images. a1-a8 are the color holograms captured by our lensfree holography setup, each with its network prediction (HER2 score), and b1-b8 are the corresponding brightfield microscopic images for the same patient tissue samples (used only for comparison).

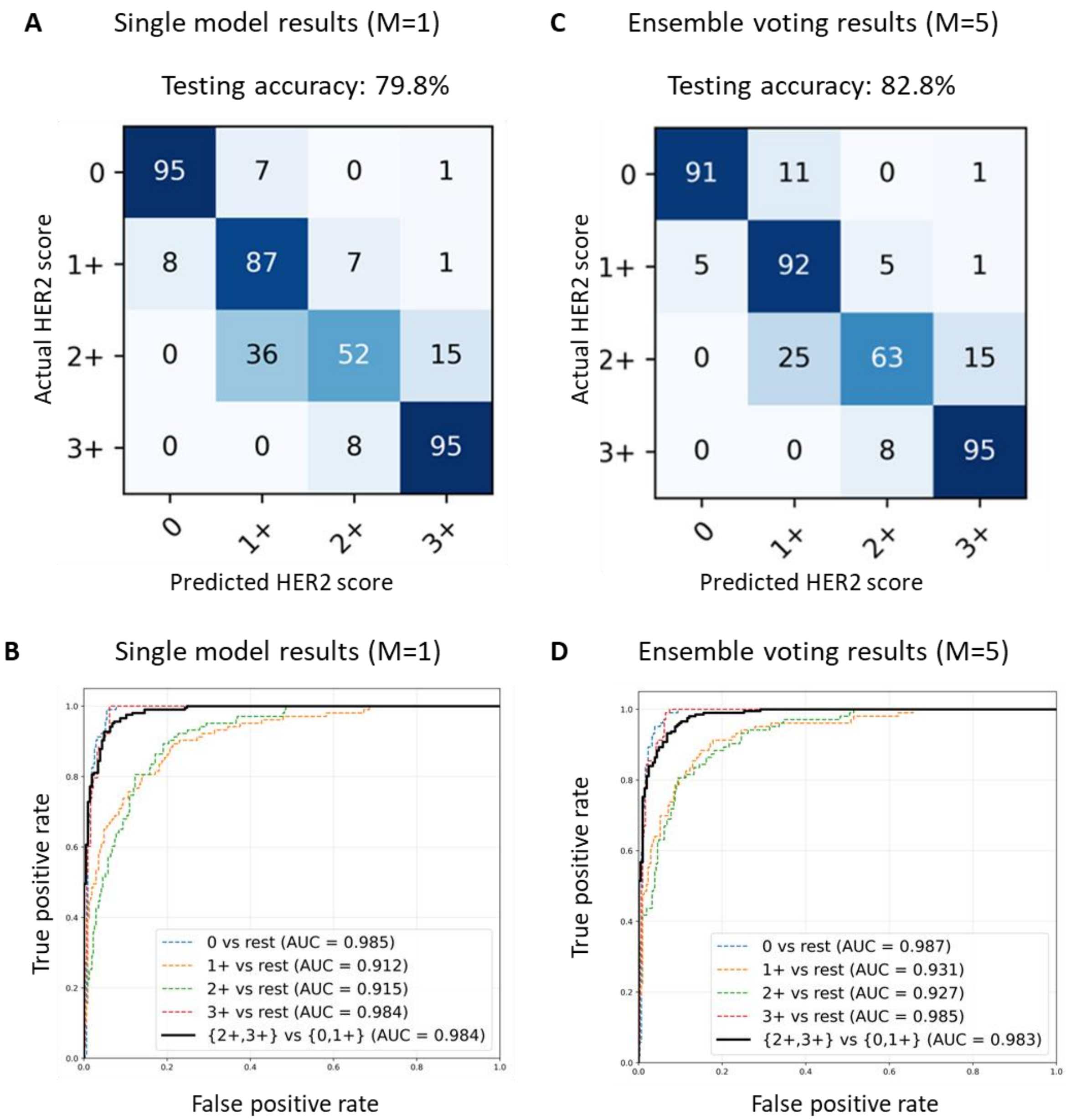

Fig. S3. Experimental results of the digital HER2 scoring using downsampled brightfield microscopy images. (A) Confusion matrix for the 4-class HER2 testing results using a single digital neural network. (B) ROC curves of the blind testing results using a single digital neural network. (C) Confusion matrix for the 4-class HER2 testing results using the ensemble voting approach with M=5 digital neural networks. (D) ROC curves of the blind testing results using the ensemble voting approach with M=5 digital neural networks.

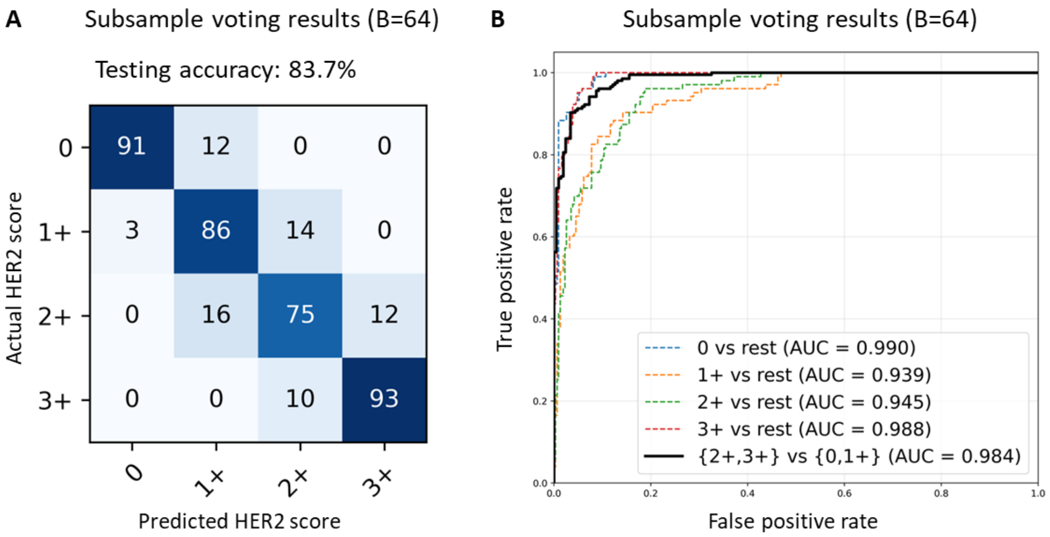

Fig. S4. Experimental results of the digital HER2 scoring using interleaved subsampled brightfield microscopy images. (A) Confusion matrix for the 4-class HER2 testing results. (B) ROC curves of the blind testing results.

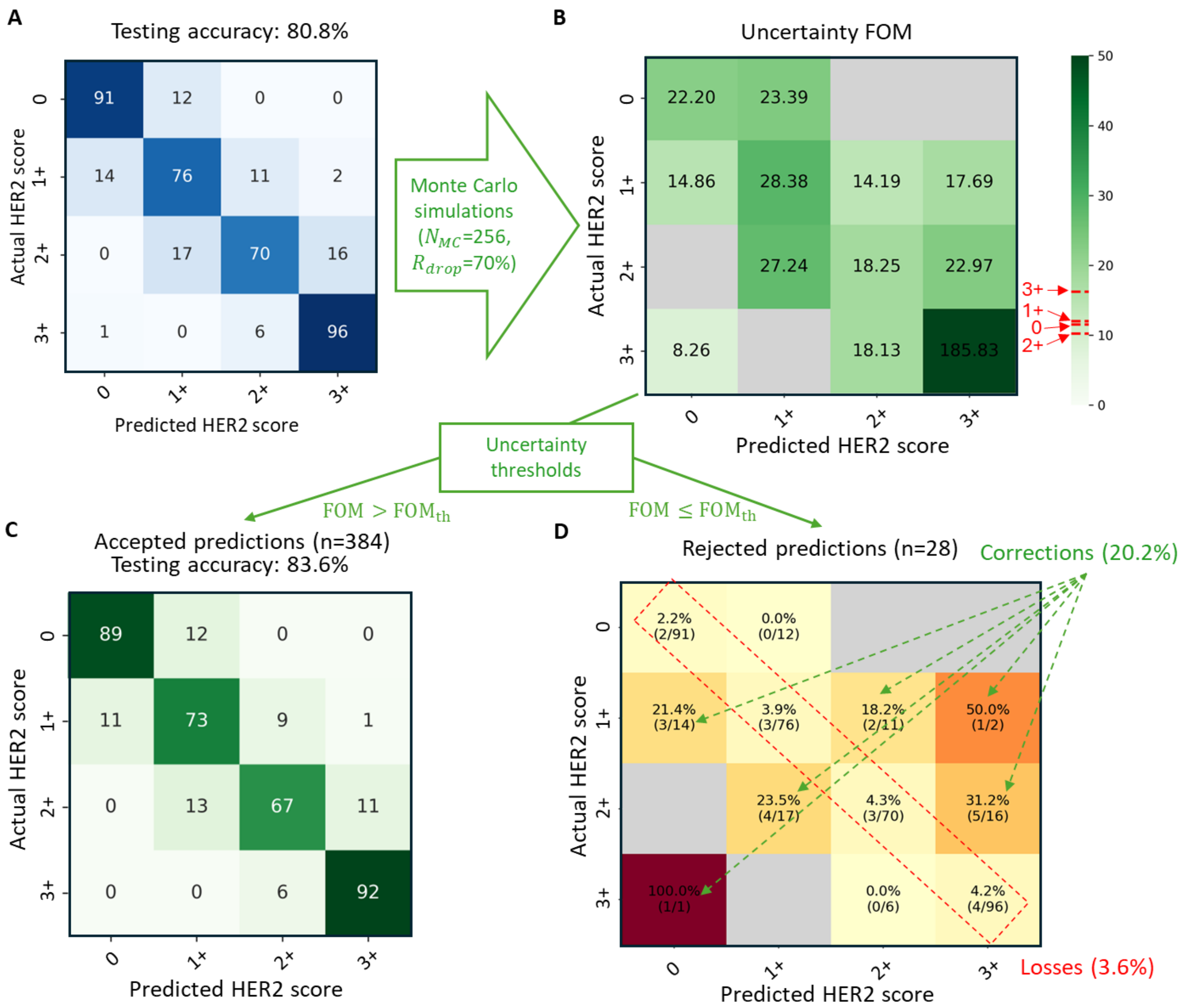

Fig. S5. Uncertainty quantification of the digital HER2 scoring using Monte Carlo (MC) dropout. (A) The original 4-class confusion matrix of the ensemble voting results from **Fig. 3c**. (B) The average uncertainty FOM for each element of the classification confusion matrix after the MC dropout-based uncertainty quantification (N_{MC}=256, R_{drop}=70%). Class-specific FOM thresholds are indicated by red dashed lines on the color bar. (C) Accepted HER2 predictions based on the uncertainty quantification, i.e., FOM > FOM_{th}. (D) Rejected HER2 predictions based on the uncertainty quantification, i.e., FOM ≤ FOM_{th}. For entries in the confusion matrix with no predictions, the corresponding value is left blank (shown in gray).

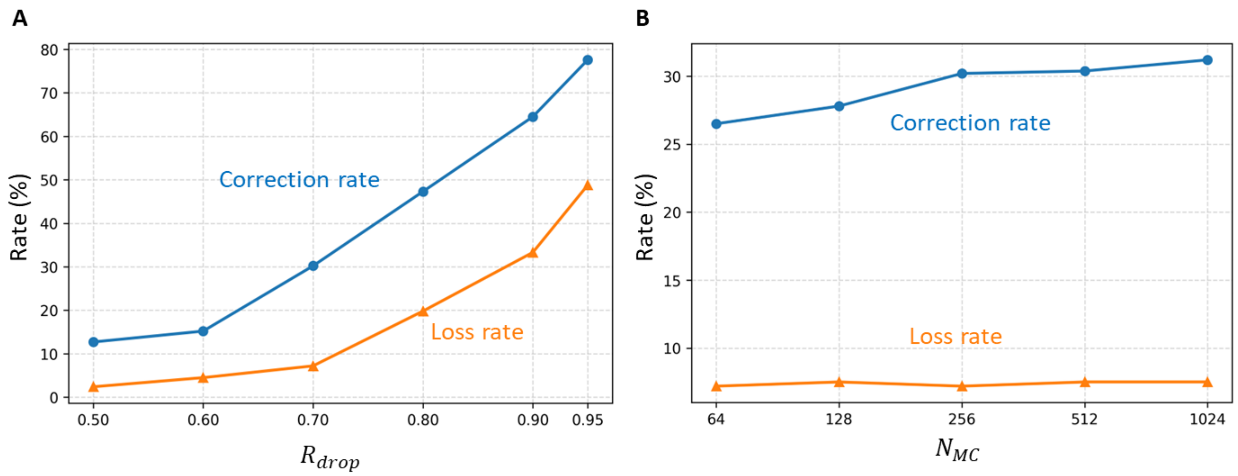

Fig. S6. Impact of MC simulation parameters on the uncertainty quantification performance. (A) Impact of the dropout rate (R_{drop}) on the correction rate and the loss rate of the uncertainty quantification strategy, illustrating the trade-off between the two. (B) Impact of the number of MC samples (N_{MC}) on the correction rate and the loss rate of the uncertainty quantification strategy.

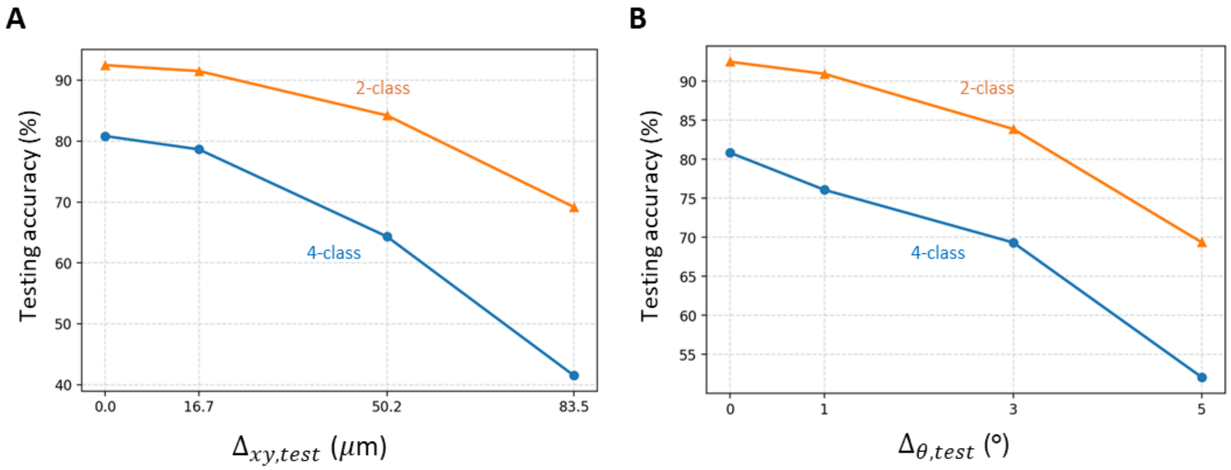

Fig. S7. Impact of image perturbations on the digital HER2 scoring performance using lensfree holography. (A) HER2 testing accuracy as a function of $\Delta_{xy,test}$. (B) HER2 testing accuracy as a function of $\Delta_{\theta,test}$.
